# Microglia Mediate Contact-Independent Neuronal Network Remodeling via Secreted Neuraminidase-3 Associated with Extracellular Vesicles

**DOI:** 10.1021/acscentsci.3c01066

**Published:** 2023-10-31

**Authors:** Corleone S. Delaveris, Catherine L. Wang, Nicholas M. Riley, Sherry Li, Rishikesh U. Kulkarni, Carolyn R. Bertozzi

**Affiliations:** † Department of Chemistry and Sarafan ChEM-H, Stanford University, Stanford, California 94305, United States; ‡ Howard Hughes Medical Institute, Stanford, California 94305, United States

## Abstract

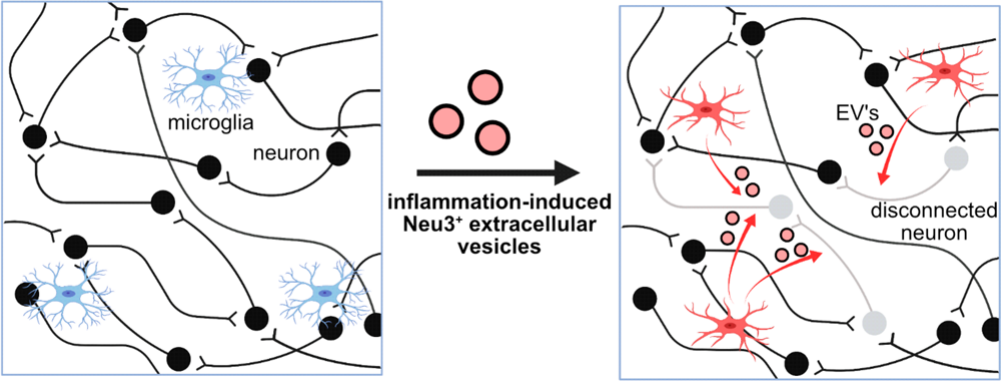

Neurons communicate with each other through electrochemical transmission at synapses. Microglia, the resident immune cells of the central nervous system, modulate this communication through a variety of contact-dependent and -independent means. Microglial secretion of active sialidase enzymes upon exposure to inflammatory stimuli is one unexplored mechanism of modulation. Recent work from our lab showed that treatment of neurons with bacterial sialidases disrupts neuronal network connectivity. Here, we find that activated microglia secrete neuraminidase-3 (Neu3) associated with fusogenic extracellular vesicles. Furthermore, we show that Neu3 mediates contact-independent disruption of neuronal network synchronicity through neuronal glycocalyx remodeling. We observe that *NEU3* is transcriptionally upregulated upon exposure to inflammatory stimuli and that a genetic knockout of *NEU3* abrogates the sialidase activity of inflammatory microglial secretions. Moreover, we demonstrate that Neu3 is associated with a subpopulation of extracellular vesicles, possibly exosomes, that are secreted by microglia upon inflammatory insult. Finally, we demonstrate that Neu3 is necessary and sufficient to both desialylate neurons and decrease neuronal network connectivity. These results implicate Neu3 in remodeling of the glycocalyx leading to aberrant network-level activity of neurons, with implications in neuroinflammatory diseases such as Parkinson’s disease and Alzheimer’s disease.

## Abstract 2

Microglia secrete the membrane-tethered glycolipid sialidase, neuraminidase-3, associated with extracellular vesicles affecting the disconnection of neuronal networks.

## Introduction

The brain is made of a vast interconnected and interdependent network of neurons, communicating information through synapses. Microglia, the resident immune phagocytes of the central nervous system, prune these synapses through several mechanisms, including direct and complement-mediated phagocytosis.^1−7^ These activities are upregulated in the context of neuroinflammatory pathologies, including Alzheimer’s disease and Parkinson’s disease.^8−10^ However, the specific mechanisms by which hyperinflammatory microglia mediate these effects remain unclear, especially in the context of how these actions impact neuronal networking and communication through synapses. Given that neurodegenerative diseases correlate with aberrant network-level neuronal activity,^9,10^ it is important to understand the molecular mechanisms by which inflammatory microglia regulate neuronal communication.

Neuroinflammation has been correlated with changes in the glycocalyx—the coating of sugars on cell surfaces—of both neurons and microglia.^11−15^ Sialic acids are a particular subset of bioactive sugars in the glycocalyx. They are known to modulate neuronal excitability and plasticity,^7,16^ and changes in the sialylation state are associated with neuroinflammation and microglial activation.^16−22^ Upon exposure to inflammatory stimuli, microglia have been observed to release sialidase activity into the surrounding media, which effects desialylation,^17,22,23^ deposition of opsonizing factors,^18,23^ microglial activation,^16,23^ and phagocytosis of neurons.^17,23^ Additionally, our lab has recently identified the sialylation state as a critical factor in maintaining neuronal excitability and network integration.^21^ Collectively, these observations point to the glycocalyx as a regulator of neuronal activity.

Herein, we tested the hypothesis that sialidases released by microglia could affect contact-independent neuronal network desynchronization. We found that the peripheral membrane glycolipid sialidase neuraminidase-3 (Neu3) is secreted by microglia upon activation by inflammatory stimuli. Neu3 was localized to a population of extracellular vesicles that are fusogenic with neurons. Using a voltage-sensing imaging dye, we found that Neu3 is both necessary and sufficient to mediate the disconnection of neuronal networks. Based on these data, we propose a mechanism in which microglia secrete Neu3 to remodel neuronal glycocalyces to modulate neuronal connectivity. These results have implications for how neuroinflammation results in neuronal network dysfunction.

## Results and Discussion

### Activated Microglia Upregulate *NEU3* and Require *NEU3* to Desialylate Neuronal Glycocalyces

**Figure 1:**
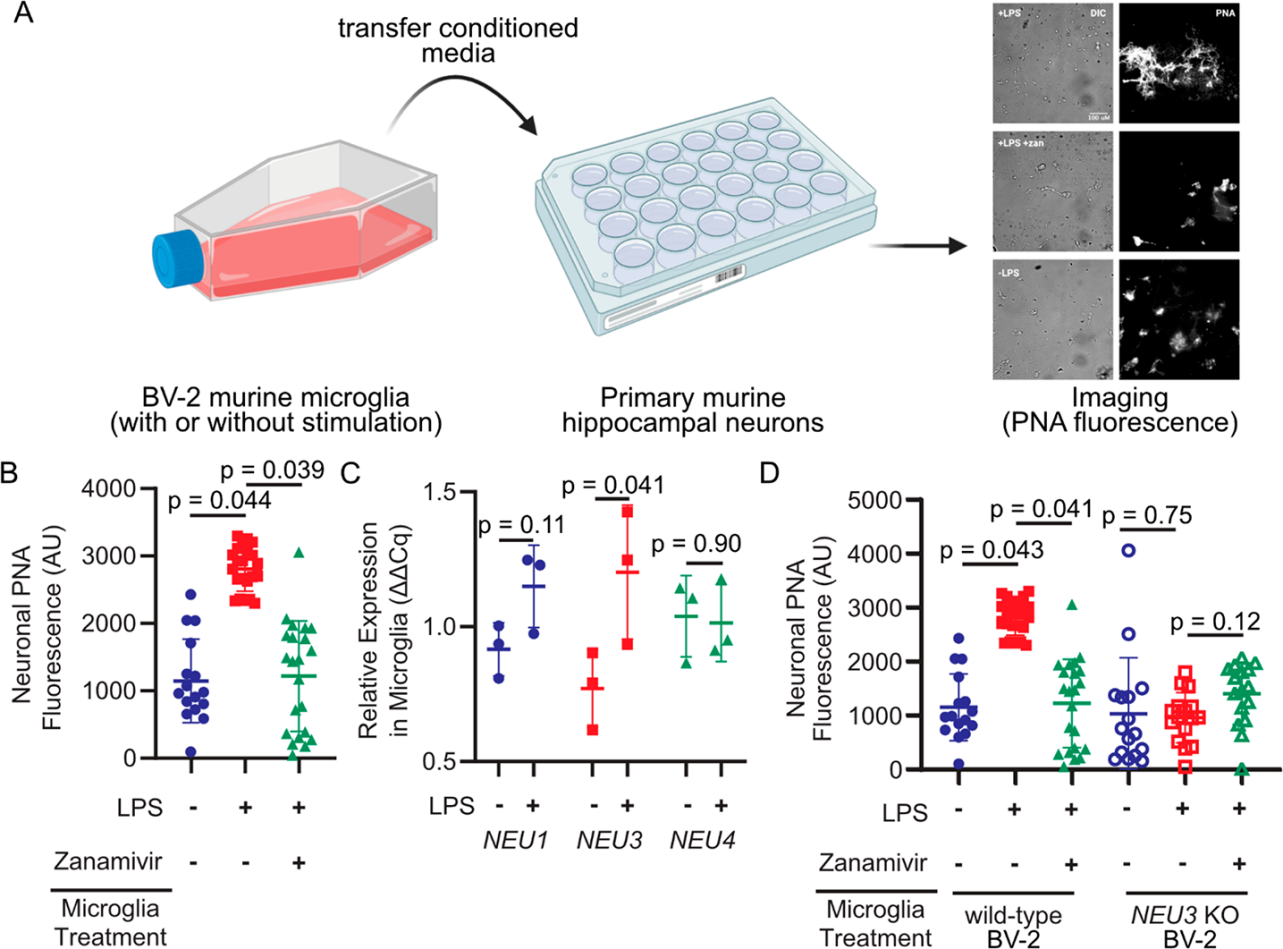
Microglia upregulate and NEU3 and active Neu3 is necessary for secreted sialidase activity. (A, B) Primary mouse hippocampal neurons were treated with conditioned media from resting or LPS-activated BV-2 microglia in the presence or absence of zanamivir. Representative scheme and images (A) and quantification of fluorescence (B) reveal that LPS-activation causes a 3-fold increase in the PNA signal compared to resting (+LPS vs −LPS, *p* = 0.044), an effect abrogated by pharmacological sialidase inhibition (−LPS vs +LPS + zan, *p* = 0.90; +LPS vs +LPS + zan, *p* = 0.039). Hypothesis testing performed with a hierarchical permutation test, *n* = 3 coverslips/condition, avg. 20 neurons/condition. (C) Quantification of transcript levels of *NEU1*, *NEU3*, and *NEU4* by qPCR in resting and LPS-activated BV-2 microglia (*NEU1*, *p* = 0.11; *NEU3*, *p* = 0.041; *NEU4*, *p* = 0.90). (D) Neurons were treated with conditioned media from wild-type (WT) or *NEU3* knockout (*NEU3* KO) BV-2 microglia with or without deoxy-2,3-anhydroneuraminic acid (DANA) and stained with peanut agglutinin (PNA). Media from activated WT microglia produced a 3-fold increase in desialylation compared to resting (−LPS vs +LPS, *p* = 0.043; +LPS vs +LPS + zan, *p* = 0.041), but media from *NEU3* KO microglia exhibited no significant change in desialylation in response to LPS or zanamivir (−LPS vs +LPS, *p* = 0.75; +LPS vs +LPS + zan, *p* = 0.12). *n* = 3 coverslips/condition, 60 total WT cells, 48 total *NEU3* KO cells. Hypothesis tests were performed with a hierarchical permutation test.

We and others have observed that microglial secretions possess sialidase activity and are capable of desialylating model cell lines^24^ and primary neurons (Figure 1a,b). Notably, these effects can be pharmacologically inhibited with zanamivir, which has inhibitory activity for human sialidases.^25^ Of the four mammalian sialidases, three have reported expression in the brain.^26^ To identify the sialidase(s) secreted by activated microglia responsible for this activity, we activated BV-2 murine microglia using lipopolysaccharide (LPS) and assessed relative mRNA expression of sialidase genes using qPCR. We observed a 50% increase in *NEU3* transcripts following activation (*p =* 0.041) and statistically insignificant changes in *NEU1* and *NEU4* (*p* = 0.11 and *p* = 0.90, respectively) (Figure 1c). To investigate the role of the glycolipid sialidase Neu3^27^ in desialylating neurons, we generated *NEU3* (the gene encoding Neu3) knockout (KO) BV-2 microglia and compared the sialidase activity of wild-type (WT) and *NEU3* KO BV-2 secretions (Figure S1). *NEU3* KO conditioned media exhibited minimal sialidase activity compared to WT (*p* = 0.043), as measured by peanut agglutinin (PNA) binding of terminal galactose residues exposed by desialylation on neuronal membranes (Figure 1d). Consistent with the observation that Neu3 is predominantly a glycolipid (e.g., ganglioside) sialidase,^27−30^ we did not observe significant changes in sialylated glycoproteins of neurons treated with conditioned media from WT versus *NEU3* KO microglia (Table S1).

As prior studies have implicated Neu1 translocation and desialylation in cis as a critical component of microglial activation,^14,15,23^ we sought to determine whether the loss of secreted sialidase activity in *NEU3* KO BV-2 cells was a consequence of impaired inflammatory activity. We observed that *NEU3* KO cells had impaired autodesialylation in response to LPS treatment compared to WT as measured by periodate labeling of sialic acids (*p* = 0.51 and = 0.038, respectively), but that both WT and *NEU3* KO cells upregulated TNFα to similar levels in (WT, *p* = 0.002; *NEU3* KO, *p* = 0.02) (Figure S2). Therefore, *NEU3* KO cells are still able to secrete inflammatory signals. TNFα secretion in both cell lines was inhibited by the pan-sialidase inhibitor deoxy-2,3-anhydroneuraminic acid (DANA), consistent with previous observations with Neu1.^14,23^ Given that the BV-2 microglia are still capable of secreting inflammatory molecules, Neu3-mediated autodesialylation is not a prerequisite for inflammatory activity in the manner as has been reported for Neu1.^15^ Moreover, these data implicate Neu3 as the secreted sialidase, rather than an upstream component.

### Microglial Secrete Neu3 Associated with Extracellular Vesicles That Fuse with Neurons

**Figure 2:**
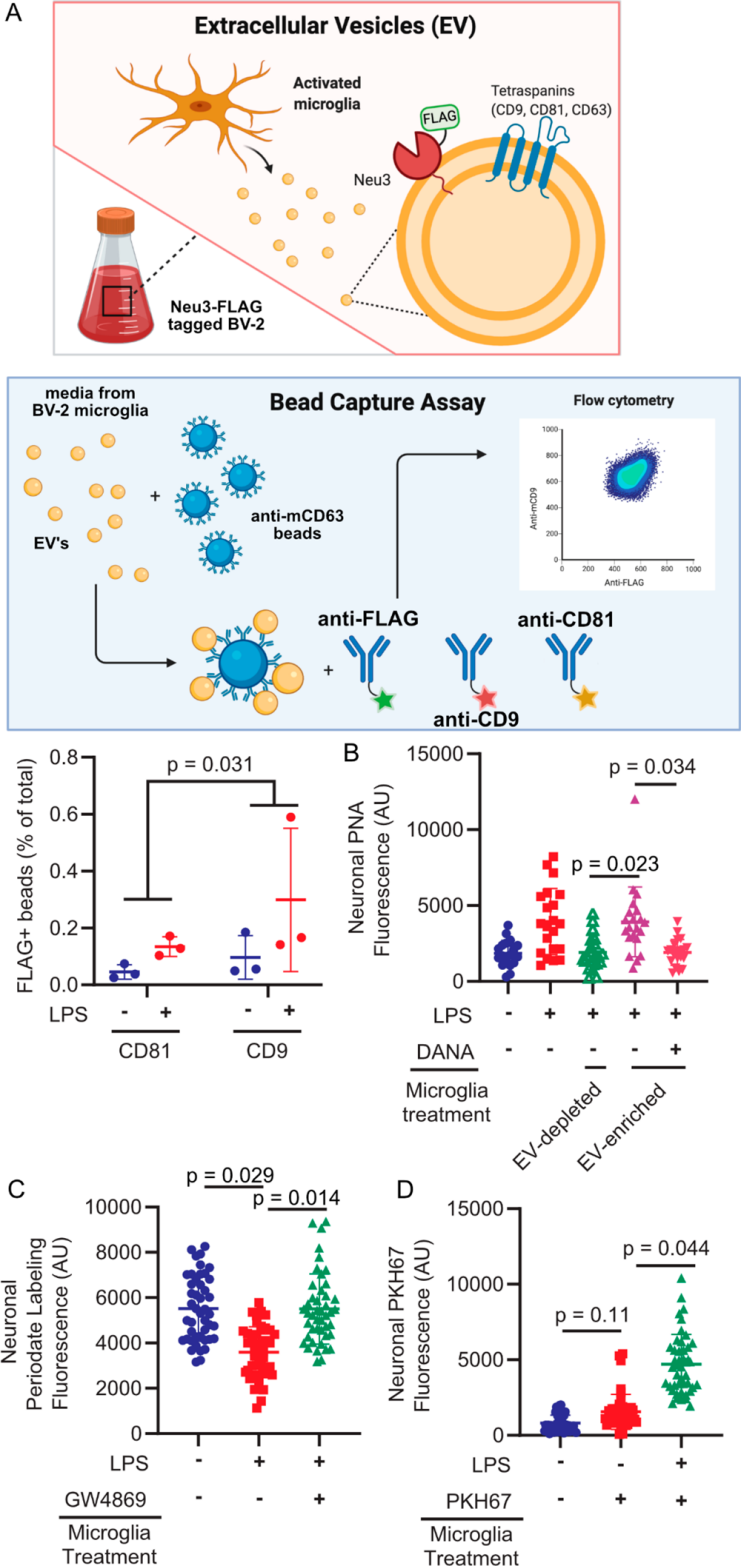
Neu3 is associated with microglia-derived fusogenic extracellular vesicles. (A) Endogenous NEU3 was FLAG-tagged in BV-2 microglia by homology-directed recombination. After exposure of BV-2 microglia with endogenously FLAG-tagged Neu3 to vehicle or LPS, EVs were captured on anti-mCD81 or anti-mCD9 coupled beads, labeled with fluorophore-coupled anti-FLAG or anti-mCD63, and analyzed by flow cytometry. Bead captured-EVs demonstrate increased FLAG signal in LPS-treated microglia compared to resting microglia, indicating that *NEU3* colocalizes with EV markers and is released via EVs upon LPS-activation (−LPS vs +LPS, *p*-value = 0.031). *n* = 3 wells/condition, 2 capture methods/well. (B) PNA staining of neuronal surfaces treated with EV-enriched or EV-depleted media of activated WT microglia reveals that the EV-enriched fraction alone has sialidase activity (EV-enriched vs EV-depleted, *p* = 0.023; EV-enriched vs EV-enriched + DANA, *p* = 0.034). *n* = 4 coverslips/condition, 7 cells/coverslip. (C) Periodate labeling of neuronal surface sialic acids reveals that pharmacologic inhibition of EV production with GW4869 abrogates sialidase activity of EV-enriched microglia media (−LPS vs +LPS, *p* = 0.027; −LPS vs +LPS + GW4869, *p* = 0.97; +LPS vs +LPS + GW4869, *p* = 0.014). *n* = 3 coverslips/condition, 135 cells total. (D) Imaging of neurons treated with PKH67-stained microglial exosome demonstrates transferal of dye from EVs to neuronal membranes (vehicle vs −LPS, *p* = 0.15; −LPS vs +LPS, *p* = 0.044). Hypothesis testing for all panels was performed using the hierarchical permutation test. *n* = 3 coverslips/condition, 15 cells/coverslip.

Neu3 has been shown to behave as a peripheral membrane protein,^31^ with recent studies demonstrating that the enzyme is S-acylated.^32^ Additionally, microglia are known to secrete extracellular vesicles (EVs) upon activation.^33^ Therefore, we hypothesized that Neu3 might be secreted in association with EVs. To investigate this hypothesis, we isolated EVs from resting and activated microglia conditioned media using commercial lectin-based isolation kits. We confirmed that we were isolating EVs based on proteomics of the surfaceome, which identified known EV proteins, but we were unable to detect Neu3 directly (Figure S3, Table S2). To confirm that Neu3 colocalizes with EVs, we inserted a 3xFLAG-tag on the endogenous NEU3 gene by homology-directed recombination (Figure S4). We then performed an immunocapture bead assay in which magnetic beads were functionalized with antimurine CD63 to capture EVs and incubated with microglia-conditioned medium. Captured EVs were analyzed with anti-FLAG and either anti-CD9 or anti-CD81, both of which are used as EV markers.^34^ We observed a distinct FLAG-positive subpopulation of EVs, the relative population of which significantly increased upon LPS-stimulation when accounting for the two different immunoprecipitation methods by a two-sample *t* test (*p* = 0.031), suggesting that microglial Neu3 is secreted via particular subsets of CD9- and CD81-positive EVs upon immune stimulus (Figures 2a and S5). Consistent with our hypotheses, the FLAG+ subpopulation of EVs is nearly absent in resting BV-2 microglia but is significantly enriched in isolates from LPS-challenged microglia. Notably, CD9 and CD81 are specific for a diverse population of various distinct subsets of small extracellular vesicles as opposed to larger bodies.^35^ The immunodetection method provides a higher sensitivity for surface proteins, which are notoriously difficult to detect by mass spectrometry without sophisticated enrichment methods due to the comparatively low abundance of surface proteins.^36^

To confirm that EV-resident Neu3 is responsible for the sialidase activity on neurons, we treated neurons with EV-enriched or EV-depleted microglia conditioned media. We observed that EV-enriched fractions demonstrated significantly increased PNA binding activity compared to EV-depleted fractions (*p* = 0.023, Figure 2b), and we observed that this PNA binding was abrogated by incubation with DANA (*p* = 0.034, Figure 2b), suggesting that EV-resident sialidases are responsible for desialylation. Consistent with this, pharmacological inhibition of extracellular vesicle production with GW4869^37^ decreased sialidase activity of the enriched fraction (*p* = 0.014, Figure 2c). Furthermore, upon staining EVs with a membrane dye, we observed robust dye transfer to neuronal membranes, indicating vesicle fusion with neurons (*p* = 0.044, Figure 2d). These data suggest a model in which microglia expel extracellular vesicles containing Neu3, which fuse with neuronal membranes and cause desialylation of the extracellular leaflet.

### Secreted Neu3 Disrupts Neuronal Network Integration in Primary Neurons

**Figure 3:**
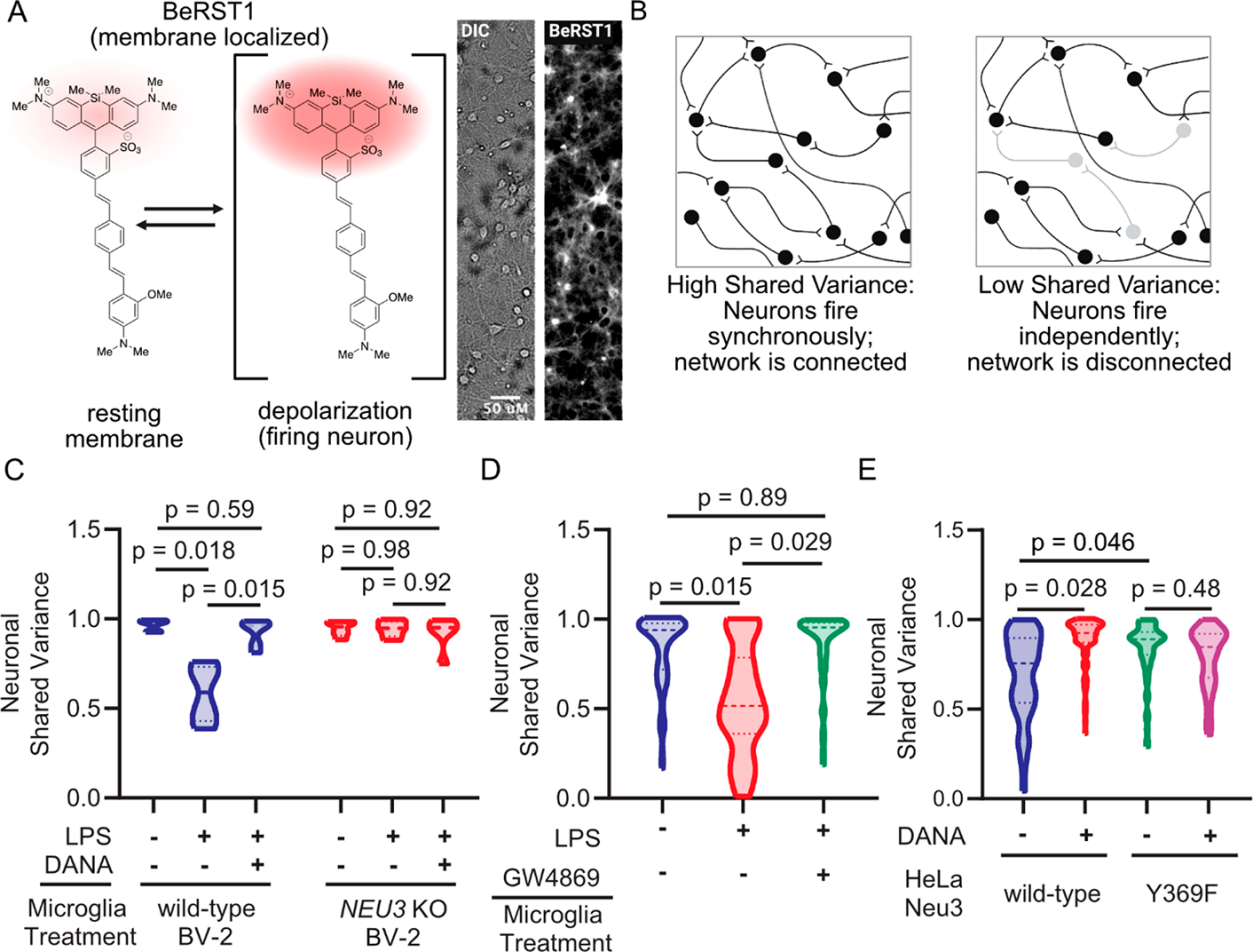
Neu3 is necessary and sufficient to disrupt neuronal network connectivity. Neurons were labeled with the voltage-sensitive dye BeRST1 and treated with extracellular-vesicle enriched media from either microglia or Neu3 overexpressing cells. Neuronal firing rates and network connectivity were analyzed by fluorescence microscopy. (A) BeRST1 is a membrane-localizing voltage-sensitive fluorophore that undergoes a dramatic increase in fluorescence intensity in response to changes in membrane potential, i.e., upon the depolarization of firing neurons. Representative brightfield and BeRST1 fluorescence of a single field of view and voltage traces of each neuron in a single field of view contain both subthreshold activity and spiking activity. (B) Network connectivity is quantitated by measuring the spike traces for individual neurons within a single field of view and then looking at the synchronicity of firing by the metric of Shared Variance. (C) EVs from conditioned media of resting or LPS-stimulated wild-type or *NEU3* KO BV-2 microglia were enriched, neurons were treated with EV-enriched media in the absence or presence of either sialidase inhibitor DANA, and neuronal activity was measured by voltage imaging with BeRST1. Treatment with EV-enriched media from wild-type BV-2 microglia results in a 38% reduction in subthreshold shared variance per neuron in −LPS vs +LPS conditions, suggesting that neurons are no longer well-connected to the network (*p* = 0.018). This effect is rescued by coincubation of EVs with sialidase inhibitor DANA (*p* = 0.015). Treatment with EVs from *NEU3* KO microglia does not have a statistically significant effect on shared variance (*p* = 0.98). (D) As in (C) but wild-type BV-2 microglia treated with or without LPS and with or without GW4869. Treatment with EV-enriched media results in a 29% reduction in subthreshold shared variance per neuron in −LPS vs +LPS conditions, suggesting that neurons are no longer well-connected to the network (*p* = 0.015). The effect is rescued by addition of GW4869 (+LPS vs +LPS + GW4869, *p* = 0.029). (E) As in (B, C), but using conditioned media from HeLa cells overexpressing either wild-type or loss-of-function (Y369F) Neu3. Treatment with EV-enriched HeLa media reveals a 15% reduction in subthreshold shared variance between WT and Y369F mutant (*p* = 0.046). Coincubation of WT EV-enriched media with DANA prevents this reduction (*p* = 0.028), while coincubation of Y369F media with DANA has no significant effect (*p* = 0.48). For (C): *n* = 3 coverslips/condition. For (D): *n* = 4 coverslips/condition, 168 neurons total. For (E): *n* = 3 coverslips/condition, 331 total neurons. All hypothesis testing was performed by hierarchical permutation tests.

Our lab has recently demonstrated that desialylation of primary neurons in culture by the highly promiscuous *Arthrobacter ureafaciens* (Au) sialidase results in decreased cell surface sialic acids and neuronal network integration.^21^ We hypothesized that Neu3 would have a similar effect. To assay this, we performed voltage imaging of primary neurons in culture using BeRST1,^38^ a membrane-localized voltage-sensitive fluorophore (Figure 3a,b). This technique enables simultaneous high-quality measurements of membrane potential in larger groups of neurons compared to traditional electrophysiology, enabling studies of network connectivity by comparing when multiple neurons in a given field of view fire.^38^ We have previously used this method in combination with factor analysis to quantify neuronal network connectivity as the “shared variance” of the network.^21^ In brief, the covariance in the firing activity of measured neurons may reflect variation in synaptic input (factors), while unexplained variance reflects the fraction of the neuron’s activity that arises spontaneously. It follows then that the ratio of shared variance to total variance measures how much of a neuron’s activity is network-driven.

Using voltage imaging, we visualized firing patterns of neurons treated with enriched EVs from wild-type or *NEU3* KO BV-2 microglia. Using factor analysis to quantify network connectivity, as we have previously described,^21^ we found that neuronal networks treated EVs from activated wild-type BV-2 microglia experienced a 38% decrease in shared variance compared to neuronal cultures treated with EVs from wild-type resting BV-2 microglia (*p* = 0.018), an effect that was abrogated by the addition of sialidase inhibitor DANA to the neuronal culture (*p* = 0.015) (Figure 3c). This indicates that the integration of measured neurons into a network had been significantly disrupted by treatment with EVs from activated wild-type BV-2 microglia and that this effect is sialidase dependent. Importantly, EVs from activated *NEU3* KO microglia did not have a statistically significant change in shared variance compared to EVs from resting *NEU3* KO microglia (*p* = 0.98) and had a 36% higher shared variance compared to neuronal cultures treated with EVs from wild-type activated BV-2 microglia (*p* = 0.015) (Figure 3c). Furthermore, we observed that the decrease in connectivity arising from treatment of neuronal cultures with purified EVs isolated from activated wild-type BV-2 microglia was rescued by pharmacological inhibition of EV biogenesis with GW4869 (*p* = 0.029) (Figure 3d). These results indicate that activated microglial EVs bearing Neu3 are necessary and sufficient to disrupt synaptic communication.

To isolate the effects of Neu3 over other potential regulatory components of microglial secreted EVs, we employed a reductionist system. Previous reports have described that *NEU3* overexpression in HeLa cells leads to the secretion of Neu3 by association with the exterior surface of microvesicles and exosomes.^39^ We hypothesized that this system could be used to isolate the effects of EV-secreted Neu3 from other potentially inflammatory components present in the secretions of activated microglia. We transiently transfected HeLa cells with plasmids encoding either wild-type Neu3 or a catalytically inactive point mutant (Y369F) (Figure S6) and enriched EVs from the conditioned media, as we did with the BV-2 conditioned media. Using periodate labeling, we observed that these EV-enriched fractions were still capable of desialyzing neuronal membranes (Figure S7). These data demonstrate that EV-associated Neu3 is sufficient to remodel neuronal glycocalyces, supported by previous studies that have shown HeLa EVs have negligible sialidase activity without *NEU3* overexpression.^39^ Using voltage imaging, we observed that while treatment of neuronal cultures with EVs isolated activated wild-type BV-2 microglia resulted in a markedly lower firing rate compared to treatment with EVs isolated from resting BV-2 microglia (−1.7 Hz, *p* = 0.048), neither wild-type nor Y369F Neu3 containing HeLa-derived EV’s caused significant changes in the firing rate between each other (*p* = 0.57) or in the presence versus absence of DANA (WT: −0.26 Hz, *p* = 0.58; Y369F: −0.14 Hz, *p* = 0.65) (Figure S8). These data indicate that the observed decrease in the firing rate of neurons treated with EVs from activated microglia is likely due to secreted factors other than Neu3.

Factor analysis of cultures treated with HeLa-derived EVs revealed a 15% decrease in per-neuron shared variance between WT and Y369F-treated cultures (*p* = 0.046), indicating a Neu3 activity-dependent loss of connectivity (Figure 3e). Congruent with this, pharmacological inhibition of Neu3 with DANA abrogated the effect of wild-type Neu3 on neuronal connectivity in culture but had no significant effect in Y369F-treated cultures (WT, *p* = 0.028; Y369F, *p* = 0.48; Figure 3e). Given that the decrease in network connectivity was only observed in the wild-type *NEU3* overexpression conditions, the decrease in network connectivity was rescued by pharmacological inhibition of sialidase activity, no DANA-dependent effect was observed in the enzymatically inactive control transfection, and all these effects were observed from EV-isolates, we can conclude that active EV-associated Neu3 alone is sufficient to drive changes in neuronal communication, a previously unknown function of Neu3.

## Conclusions

The prototypical sialic acid, 5-*N*-acetylneuraminic acid, was named based on the observed abundance of sialic acids on the external leaflet of neurons, particularly sialylated glycolipids known as gangliosides.^40^ Neu3 is a membrane-associated glycolipid sialidase^28^ that we speculated might play a role in regulating neuronal connectivity. The data herein present a new mechanism by which microglia regulate neuronal sialylation by secretion and transfer of Neu3 via extracellular vesicles. Moreover, we show that Neu3-mediated remodeling has a dramatic impact on the connectivity of neuronal networks, providing molecular detail for a contact-independent regulation pathway of neuronal network synchronicity. These findings demonstrate a novel axis by which microglia and neurons communicate. Indeed, sialoglycans may serve as a mechanistic bridge among neuroinflammation, neuronal pruning, and downstream changes in electrophysiology, which would position them as potential therapeutic targets for neurological disorders. The electrical mechanism of this rewiring, as well as other neuroinflammatory signals that lead to this effect, is exciting grounds for future research.

## Supporting Information Available

Materials and methods used in this work; tables of oligonucleotides and gene fragments used in this work; Tracking of Indels by Decomposition (TIDE) analysis of *NEU3* KO BV-2 microglia; Neu3-mediated autodesialylation of BV-2 microglia; full Western blots; representative cytograms; representative spike traces and fluorescence microscopy images. Supplementary tables containing sialoglycan-enrichment based proteomics and extracellular vesicle surface proteomics data (PDF)
